# Screening and regulatory mechanisms of biomarkers related to neddylation in laryngeal squamous cell carcinoma

**DOI:** 10.3389/fmolb.2025.1654064

**Published:** 2025-10-22

**Authors:** Xin Wang, Haixiang Zhang, Jinping Wang

**Affiliations:** ^1^ Department of Otolaryngology, Shaanxi Provincial People’s Hospital, Xi’an, Shaanxi, China; ^2^ Shaanxi Provincial Key Laboratory of Infection and Immune Diseases, Shaanxi Provincial People’s Hospital, Xi’an, Shaanxi, China

**Keywords:** laryngeal squamous cell carcinoma, neddylation, biomarkers, LSCC risk model, WSB2

## Abstract

**Objective:**

Neddylation is a crucial posttranscriptional modification involved in tumor progression. This study aimed to explore neddylation-associated biomarkers and the underlying mechanism in laryngeal squamous cell carcinoma (LSCC).

**Methods:**

This study evaluated the expression of neddylation-related genes (NRGs) retrieved from the Reactome and TCGA databases to conduct a series of analyses and constructed an LSCC prognostic risk model followed by functional enrichment and mechanism prediction. Moreover, the key genes involved in this signature were also confirmed in an *in vitro* cell model.

**Results:**

A total of 79 NRGs were differentially expressed in LSCC (*P*.adj <0.05). A prognostic gene signature was constructed, and *COMMD2*, *WSB2* and *CUL9* were determined to be prognostic genes. The nomogram indicated that this gene signature performed well in forecasting the 1-, 3-, and 5-year overall survival of LSCC patients. The *CUL9* and *WSB2* genes were enriched in RIBOSOME, and silencing WSB2 significantly inhibited the malignant behaviors of LSCC cells. In this gene signature, patients could be markedly distinguished into high- and low-risk groups characterized by different immune infiltration and drug sensitivity between them. WSB2 and COMMD2 jointly predicted that hsa-miR-185-5p, hsa-miR-4644 and hsa-miR-4306 were the common microRNAs (miRNAs) and regulatory networks.

**Conclusion:**

This study successfully established a neddylation-associated prognostic risk model for LSCC and revealed that COMMD2, WSB2, and CUL9 could act as new therapeutic targets, which might provide valuable information for the research and treatment of LSCC.

## Introduction

Laryngeal squamous cell carcinoma (LSCC), mostly arising from the laryngeal mucosal epithelium is the 2nd most frequent cancer in affecting the head and neck. The clinical manifestations of LSCC typically include dyspnea and dysphagia. Factors such as human papillomavirus infection and alcohol abuse contribute to the rapid progression of LSCC, resulting in persistently high morbidity and mortality rates (Xiang et al., 2021; Li et al., 2022). In 2018, there were 177,422 newly reported cases of LSCC, with 154,977 cases in males and 22,445 in females. The total number of deaths that year was 94,771, with 68,841 more males than females. Even worse, there were 180,000 new cases and nearly 100,000 deaths from LSCC worldwide by 2020 (Li et al., 2022; Siegel et al., 2019; Bradford et al., 2020; Bray et al., 2018). Current treatment modalities for LSCC include chemotherapy, radiotherapy, and conservative surgical interventions. Recent advancements have introduced enhanced immunotherapies and targeted therapies, including platinum-based chemotherapeutic agents, which are commonly used in LSCC management (Almadori et al., 2005; Ahn et al., 2022; Manevich et al., 2022; Cramer et al., 2019). However, despite these therapeutic developments, early diagnosis, survival rates, and patient prognoses for LSCC remain suboptimal. These persistent challenges can be attributed to several factors, such as local invasion, high metastatic potential, chemotherapy resistance, and the lack of effective biomarkers that elucidate the molecular mechanisms of LSCC (Gao et al., 2020; Xue et al., 2019). Consequently, there is an urgent need for significant improvements in both the treatment and prognosis of LSCC.

Neddylation, a novel biological process of protein posttranslational modification, involves a three-step enzyme-mediated reaction that covalently attaches the ubiquitin-like molecule NEDD8 (neuronal precursor cell-expressed developmentally downregulated protein 8) to its substrates, primarily members of the cullin protein family (Enchev et al., 2015; Mao et al., 2023; Zhang S. et al., 2024). Neddylation overactivation has been shown to lead to the degradation of tumor suppressor factors, which can induce apoptosis and contribute to carcinogenesis. This mechanism is critical for regulating various biological functions, particularly within the tumor microenvironment (Gonzalez-Rellan et al., 2023; He et al., 2023; He et al., 2022; Zhou et al., 2019). MLN4924 (pevonedistat), a neddylation inhibitor, suppresses the progression of head and neck squamous cell carcinoma (HNSC) and sensitizes the carcinoma cells to ionizing radiation (Vanderdys et al., 2018). Combination of MLN4924 further enhances the chemotherapeutic efficacy of (S)-10-hydroxycamptothecin (10-HCPT) in HNSC via activating NFKB1 pathway (Gu et al., 2023). Neural precursor cell expressed developmentally downregulated protein 9 (NEDD8) is an ubiquitin-like protein to mediate covalently conjugation to a lysine residue of substrate proteins during neddylation. It is reported that MLN4924 inhibits the progression of HNSC via NEDD8/CUL4/TSC2 axis mediating inactivation of mTOR signaling pathway (Jiang et al., 2024). Targeting inhibition of CUL4A synergizes of carcinoma to cisplatin via DDB2-dependent pathway, thereby promoting the long-term prognosis (Jones et al., 2022). These evidences suggested that targeting neddylation represents a promising strategy for HNSC treatment. However, the role of neddylation-related biomarkers in LSCC was still uncertain.

In the current study, we employed bioinformatics approaches, including transcriptome sequencing data, to identify biomarkers associated with neddylation in LSCC, followed by exploration of potential regulatory pathways and mechanisms. In addition, the bifunctional role of key biomarkers was validated via *in vitro* assays. These analyses provide insights into the diagnosis, treatment, and prognosis of LSCC.

## Materials and methods

### Data sources

Pathway enrichment analysis was performed using the Reactome database (https://reactome.org/). The ‘Pathway Browser’ for *Homo sapiens* was queried with the keyword “neddylation,” and results were filtered by selecting the “Molecules” category to retrieve neddylation-related pathways and molecules. All pathways and reactions identified through this search underwent manual review and screening to ensure direct relevance to the neddylation process. A total of 246 neddylation process associated genes were downloaded from the Reactome database on 5 July 2024. These carefully curated genes were designated as neddylation-related genes (NRGs). A total of 115 LSCC patients contained in the HNSC dataset of The Cancer Genome Atlas (TCGA) were screened in this study. Then, the RNA-Seq data along with the patient information of them were downloaded in FPKM format from the official GDC Data Portal using TCGAbiolinks and named TCGA-LSCC, including the transcriptional data, somatic mutation data, clinical characteristics and survival information.

### Differential expression gene analysis

In the TCGA-LSCC dataset, differential analysis between the LSCC tumor (*n =* 12) and normal samples (*n =* 115) was performed using the “DESeq2” (v 1.46.0) (Love et al., 2014) package in R software (v 4.2.2). The differentially expressed genes (DEGs) were selected with the thresholds of |log_2_fold change (FC)| >0.5 and *P*.adj (Benjamini–Hochberg method) <0.05. Moreover, DEGs and the top 10 most significantly upregulated and downregulated genes were visualized by a volcano plot and heatmap using “ggplot2” in R, respectively.

### DEG enrichment and protein‒protein interaction (PPI) network analyses

Gene Ontology (GO) and Kyoto Encyclopedia of Genes and Genomes (KEGG) pathway enrichment analyses on the DEGs were conducted using clusterProfiler (Yu et al., 2012) packages in R to identify the common functions and related pathways of the DEGs. The R package “ggplot2” was used to draw bar charts of the top 10 biological processes (BP), molecular functions (MF), cellular components (CC), and KEGG pathways significantly enriched by DEGs with a threshold of *P*.adj <0.05 (Benjamini–Hochberg method). The “ggvenn” (v 0.1.10) package in R was employed to screen and draw the gene intersection diagram. Finally, Cytoscape (v 3.9.1) (Shannon et al., 2003) was utilized to visualized the PPI network diagram (Interaction score ≥0.4).

### Construction of the risk score model

Based on the calculated risk scores, LSCC associated risk model was established via univariate Cox and least absolute shrinkage and selection operator (LASSO) regression analyses. The survival (v 0.5.0) tool was used for univariate analysis, and the “glmnet” (v 4.1.8) tool was applied for LASSO regression analysis. The parameter family was set as Cox (HR ≠ 1, p > 0.05) to implement LASSO logistic regression. The graphs of gene coefficients and the error graph of 10-fold cross-validation were obtained by selecting strongly correlated features. When the model error was minimized, the optimal model and the corresponding lambda (λ) value were determined.

### Evaluation and validation of the prognostic risk signature

To evaluate and verify the prognostic risk signature, we first computed the risk score via the following equation:

$$\text{Risk }score=\sum_{\left(i=1\right)}^{n}Coef\left(i\right)\times \text{expr}\left(i\right)$$

where Coe*f* denotes the risk coefficient associated with each gene, while expr signifies the expression level of each gene. The “ggplot2” (Gustavsson et al., 2022) in R was employed to depict the survival curves and survival status of patients with high- and low-risk. The “survminer” package was used to depict survival curves for overall survival (OS) between the high- and low-risk groups (p < 0.05).

### Risk signature evaluation

Based on the risk signatures obtained from aforementioned, the “survivalROC” package (v 1.0.3.1) was used to draw receiver operating characteristic (ROC) curves of 1-, 3- and 5-year survival time point. The ROC curve plots two parameters: the true positive rate and the false positive rate. The area under the curve is called the area under the curve (AUC), which is used to represent the prediction accuracy and sensitivity. The higher the AUC value is, that is, the larger the area under the curve is, the higher the prediction accuracy. According to the results of the prognostic genes, the R package “rms” (v 7.0.0) (https://github.com/harrelfe/rm) was utilized to build a nomogram for the 1-, 3-, and 5-year survival rates of LSCC patients. In addition, the “timeROC” package (v 0.4) (Blanche et al., 2013) was utilized to draw the corresponding ROC curves to assess the prognostic ability. The “ggDCA” R package (v 1.2) was used for decision curve analysis (DCA) to evaluate the clinical benefit of this model.

### Gene set enrichment analysis (GSEA)

The gene‒gene interaction network (GGI) of the prognostic genes was established via the GeneMANIA website. The “c2.cp.kegg.v2023.1.Hs.symbols.gmt” gene set downloaded from the MSigDB database was used as the background gene set. Spearman correlation analysis was performed between each prognostic gene and all the other genes to obtain correlation coefficients (p < 0.05). These coefficients were then sorted from small to large. On the basis of the sorting results, the top 5 genes were selected for display, and GSEA was performed on the prognostic genes.

### Immune microenvironment analysis

“GSVA” (Hänzelmann et al., 2013) package (v 2.0.1) in R was used to compute the infiltration condition of immune cells in LSCC patients in the training set on the basis of the single-sample GSEA (ssGSEA) algorithm. A heatmap was drawn to observe the distribution proportions of 28 types of immune cells between different groups. ssGSEA was used to estimate the samples in the training set. With the immune-related genes provided in the article by Charoentong et al. (2017) as the background gene set, enrichment scores of immune cells were obtained, and the Wilcoxon rank-sum test was performed (p < 0.05). Spearman correlation analysis was carried out, and the results with a correlation of |R| > 0.3 and *p* < 0.05 were considered to have a significant correlation.

### Drug sensitivity exploration

To further evaluate the associations between the high- and low-risk groups and the response to chemotherapy drugs, in the TCGA-LSCC training set patients, the R package “pRRophetic” (v 0.5) (Geeleher et al., 2014) was used to calculate the IC50 values of 138 common chemotherapies and molecularly targeted drugs for all patients. IC50 is the half-maximal inhibitory concentration. Generally, the IC50 is used to measure the cytotoxicity of a drug or the degree of drug tolerance of the cell. The lower the IC50 is, the stronger the performance of the drug (p < 0.05).

### Molecular regulatory network analysis

To explore the microRNAs (miRNAs) that regulate the prognostic genes, the miRTarBase database (https://mirtarbase.cuhk.edu.cn/) were applied to jointly forecast the common miRNAs of the prognostic genes. Then, the targeted miRNA‒mRNA regulatory network was visualized by Cytoscape. WSB2 specific associated miRNAs expression was validated using the ENCORI database in HNSC (https://rnasysu.com/encori/panMirDiffExp.php) on 10 September 2025. The expression of downregulated miRNAs in HNSC was verified in LSCC cells lines with HaCaT as control.

### Cell culture and transfection

The human LSCC cell line TU-138 and immortalized epidermal HaCaT cell line were acquired from Xiamen Immocell Biotechnology Co., Ltd. (Xiamen, Fujian, China), and the LSCC cell line TR-LCC-1 was obtained from Wuhan Pricella Biotechnology Co., Ltd. (Wuhan, Hubei, China). Specifically, HaCaT cells were maintained in DMEM, and TU-138 and TR-LCC-1 cells were maintained in RPMI 1640 (Cat. No. IMC-202-2; Xiamen Immocell Biotechnology Co., Ltd.) at 37 °C with 5% CO_2_ in the atmosphere. All media were supplemented with 10% fetal bovine serum (FBS; Cat. No. S615JY; Shanghai Basal Media, Shanghai, China) and 1% penicillin‒streptomycin. For the gene silencing experiments, siRNAs were designed using DSIR (http://biodev.extra.cea.fr/DSIR/DSIR.html). The mimic and negative control (NC) were designed according to the sequence provided in miRDB (https://mirdb.org/cgi-bin/target_detail.cgi?targetID=3460921). The details of sequences were summarized in Supplementary Table S1, and produced by GenePharma (Shanghai, China) and transfected into LSCC cell lines via Lipofectamine 3000 (Cat. No. L3000015; Invitrogen, Carlsbad, CA, United States) according to the manufacturer’s protocol. Transfection for 48 h, the cells were collected for the experiment analysis.

### Luciferase reporter assay

The wildtype (WT) and mutant (MUT) WSB2 3′UTR sequences were synthesized and cloned downstream of the luciferase reporter gene by Beijing Tsingke Biotech Co., Ltd. (Beijing, China). Then, these reporter plasmids were co-transfected with either hsa-miR-6507 mimic or NC in TU138 and TR-LCC-1 cells, respectively. After 48 h, the luciferase activity was measured using the Dual Luciferase Assay Kit (Cat. No. E2920; Promega, United States) following the manufacturer’s protocol.

### Cell viability

Cell viability was estimated via the CCK-8 assay. In detail, cells were transfected with siRNA and inoculated into 96-well plates with 5.0 × 10^3^ per well density. After culturing, 10 μL of CCK-8 solution (Cat. No. RM02823; Abclonal, Wuhan, Hubei, China) was appended to each well at 24, 48, and 72 h and hatched at 37 °C for 1 h. Then, the optimal density of each well was detected at 450 nm via a microplate reader (Thermo Fisher Scientific, United States).

### Colony formation assay

The proliferation of the cells was determined via colony formation analysis. Briefly, cells were seeded into 6-well plates after transfection at a density of 500 per well. The cells were subsequently cultured at 37 °C for 14 days, after which the medium was removed every 2 days. After culture, the medium was carefully removed, and the cells were immobilized with 4% paraformaldehyde (PFA; Cat. No. G1101-500ML; Servicebio, Wuhan, Hubei, China) and stained with 0.5% crystal violet (Cat. No. G1014-50ML; Servicebio). Finally, the staining results were imaged and calculated for further analysis.

### Wound healing assay

The migration of cells was assessed via a wound healing assay. Briefly, cells were seeded into a 6-well plate and maintained at 37 °C overnight. When the confluency reached more than 80%, a scratch was made on the cell monolayer via a 200 μL pipette. After washing with PBS to remove the suspended cells, a fresh medium was added and cultured for 48 h. During culture, the cells were pictured at 0 and 48 h to record the wound distance.

### Transwell assay

After transfection, cells were inoculated into the upper part of a transwell plate coated with 10% Matrigel in RPMI 1640 medium. Moreover, 600 μL of RPMI supplemented with 20% FBS was added to the lower chamber. After 48 h of coculture, the transwell membrane was immobilized with 4% PFA and stained with 0.5% crystal violet (Servicebio). The staining results were then imaged under an inverted microscope (Leica), and five random fields of view were counted for statistical analysis.

### Quantitative real-time PCR (qRT‒PCR)

After transfection, total RNA was isolated from the cells with RNAiso Easy (Cat. No. TCH022; Takara, Dalian, China). After quantification with a Nanodrop 2000 (Thermo, United States), 2 μg of total RNA was subjected to cDNA synthesis via the PrimeScript RT reagent Kit (Cat. No. RR047Q; Takara). Then, amplification of WSB2 and GAPDH was performed via SYBR Green (Cat. No. RK21203; Abclonal), and the relative expression of WSB2 was computed via the 2^−ΔΔCt^ method. The primers were designed and summarized in Supplementary Table S2.

### Western blotting

Proteins in cell samples were extracted using the RIPA lysis buffer (Cat. No. P0013B, Beyotime, Shanghai, China) supplemented with protease inhibitor cocktail (Cat. No. RM02916, Abclonal). After quantified using the BCA method (Cat. No. P0009, Beyotime), 25 μg of each sample was subjected for 10% SDS-PAGE and transferred onto polyvinylidene fluoride membrane (Cat. No. HVHP01300, Millipore, MA, United States). Following this, membrane was blocked with 5% skim milk for 30 min at room temperature, and then incubated with anti-WSB2 (Cat. No. D123580, Sangon, Shanghai, China) or anti-GAPDH (Cat. No. A19056, Abclonal) antibody at room temperature for 1 h. Then, membrane was incubated with anti-rabbit antibody (Cat. No. AS014, Abclonal) at room temperature for 1 h and visualized using a high sensitive ECL luminescence reagent (Cat. No. C500044, Sangon). Finally, the detected protein bands were quantified using ImageJ (v 1.54i, NIH, Bethesda, MD, United States), and compared between groups.

### Statistical analysis

Statistical analyses were performed via R Studio. The Wilcoxon test was used to assess group variance, whereas Spearman correlation analysis was applied to explore the relationships between biomarkers and immune cells. The experiment data was analyzed using the GraphPad Prism (v 9.5.0, GraphPad Software lnc., Boston, MA, United States). All the experiments were performed in triplicate independently, and presented with mean ± standard deviation. *P* < 0.05 was considered statistically significant.

## Results

### Identification of NRG-related DEGs between LSCC and normal tissues

**FIGURE 1:**
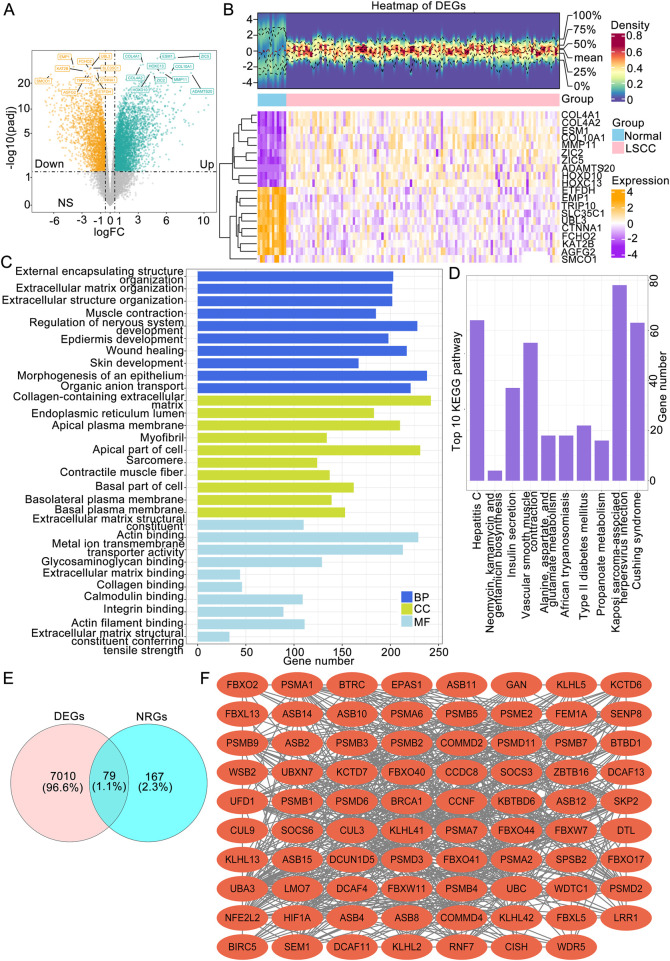
Identification of LSCC-associated NRGs followed by functional enrichment and protein interaction analyses. **(A)** Volcano plot of LSCC-associated DEGs. **(B)** Expression heatmap (B) of the top 20 DEGs between the LSCC and normal groups. **(C)** The top 10 BP, MF and CC terms of the GO and KEGG pathways enriched with DEGs. **(D)** The genes intersecting the DEGs and NRGs. **(E)** PPI network of NRG-associated DEGs. LSCC, laryngeal squamous cell carcinoma; DEGs, differentially expressed genes; NRGs, neddylation-related genes; GO, Gene Ontology; BP, biological process; MF, molecular function; CC, cellular component; KEGG, Kyoto Encyclopedia of Genes and Genomes.

Comparison analyses revealed a total of 7,089 DEGs between LSCC and normal tissues, comprising 4,377 upregulated genes and 2,712 downregulated genes (Figure 1A). Hierarchical clustering of the 20 most significant DEGs revealed distinct intergroup patterns in the expression profiles (Figure 1B). Additionally, systematic annotation of these DEGs through GO and KEGG pathway analyses revealed significant functional associations. A graphical representation of the top 10 enriched terms across biological processes (866 terms), cellular components (131 terms), and molecular functions (80 terms) is provided (Figure 1C; Supplementary Table S1), with primary enrichment observed in extracellular matrix organization, structural constituents of extracellular matrices, and collagen-associated compartments (*P*.adj <0.05). Concomitant KEGG pathway analysis revealed that 33 signaling cascades, particularly those governing muscular cytoskeletal dynamics, calcium-mediated signal transduction, and extracellular matrix-receptor crosstalk, were statistically significant (*P*.adj <0.05) (Figure 1C). To obtain LSCC associated neddylation genes, a systematic overlap analysis was performed between 246 neddylation-related genes (NRGs) derived from the Reactome database and 7,089 DEGs associated with LSCC, identifying 79 NRG-associated DEGs with potential regulatory significance (Figure 1D). A PPI network was subsequently constructed via the STRING database (interaction score ≥0.4) to investigate potential functional interactions among the 79 candidate genes, identifying 732 significant interactions among the 79 protein nodes (Figure 1E).

### Identification of a neddylation-related prognostic signature in LSCC patients

**FIGURE 2:**
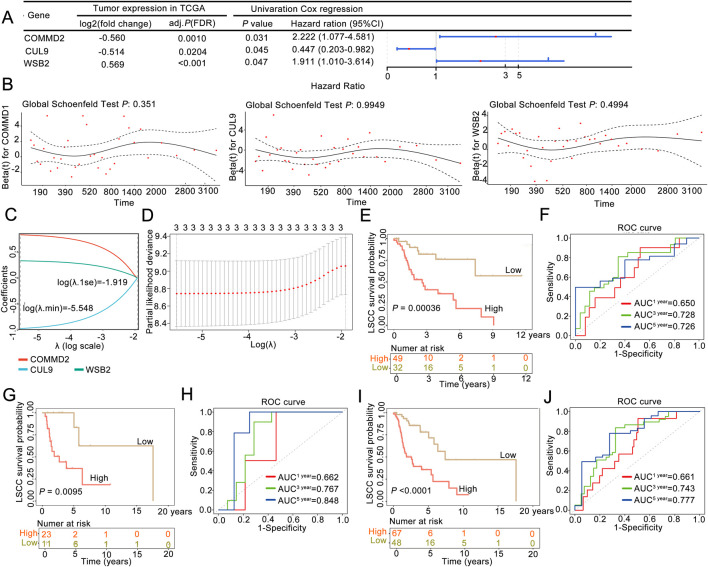
A neddylation-associated prognostic signature could be used for 3- and 5-year overall survival prediction in patients with LSCC. **(A)** Univariate Cox regression analysis. **(B)** Proportional hazards assumption test. **(C,D)** LASSO regression analysis was used to obtain the risk signature. **(E)** Kaplan‒Meier (K–M) survival curve of the training set. **(F)** ROC curve in the training set. **(G)** K‒M survival curve of the validation set. **(H)** ROC curve in the validation set. **(I)** K‒M survival curve of the whole sample. **(J)** ROC curve of the whole sample.

Firstly, we randomly divided the 115 disease samples in the TCGA-LSCC dataset into a training set and a validation set in a 7:3 ratio. Specifically, 7 represents the training set with 81 samples, which were used for the construction of the prognostic model, while 3 represents the validation set with 34 samples, which were used for the validation of the prognostic model. Next, our study integrated 79 candidate gene expression profiles with OS data to construct a neddylation-related prognostic signature in the training cohort (*n =* 81). Initially, univariate Cox regression identified three survival-associated genes (*P* < 0.05) from among the 79 candidate neddylation-related DEGs, with *COMMD2* and *WSB2* exhibiting hazard ratios (HRs) >1 (prognostic risk factors) and *CUL9* showing an HR < 1 (prognostic protective factor) (Figure 2A). To ensure the robustness of the identified prognostic genes and minimize false positives, proportional hazards (PH) assumption testing was conducted. All three genes demonstrated stable associations with survival (*P* > 0.05; Figure 2B), supporting their validity. Subsequently, LASSO regression analysis at log (λ.min) = −5.548 yielded a three-gene prognostic signature comprising *COMMD2*, *WSB2*, and *CUL9* (Figures 2C,D).

According to this three-gene signature, the prognostic risk score of each LSCC patient in the training set was calculated, and the optimal cutoff value (risk score = 0.8155) was determined via ROC analysis. With this cutoff value, patients were stratified into high-risk (*n =* 49) and low-risk (*n =* 32) groups. The survival curve and status analyses suggested that the risk scores distinguished the low- and high-risk groups in the training set (Supplementary Figure S1A), validation set (Supplementary Figure S1B), and overall set (Supplementary Figure S1C). K‒M analysis revealed that LSCC patients in the high-risk group had significantly poor OS than the low-risk group (*P* = 0.00036; Figure 2E), a finding that was consistently validated in the validation cohort (*P* = 0.0095; Figure 2G). Additionally, ROC analysis was performed to evaulate the predictive ability of the prognostic signature, yielding AUC values of 0.728 (3-year) and 0.726 (5-year) in the training set (Figure 2F) and 0.767 (3-year) and 0.848 (5-year) in the validation set, confirming the model’s good performance (Figure 2H). Finally, the three-NRG prognostic signature was validated in the overall cohort, which revealed significantly worse OS in high-risk group than in low-risk group (*P* < 0.05; Figure 2I). The AUC values for the 3-year and 5-year ROC curves were 0.743 and 0.777, respectively (Figure 2J). These findings demonstrated that the three-NRG prognostic signature effectively stratified LSCC patients into distinct risk groups and could be used for 3- and 5-year prognosis prediction, highlighting its potential clinical utility.

### Association between the risk score and clinical features of LSCC

**FIGURE 3:**
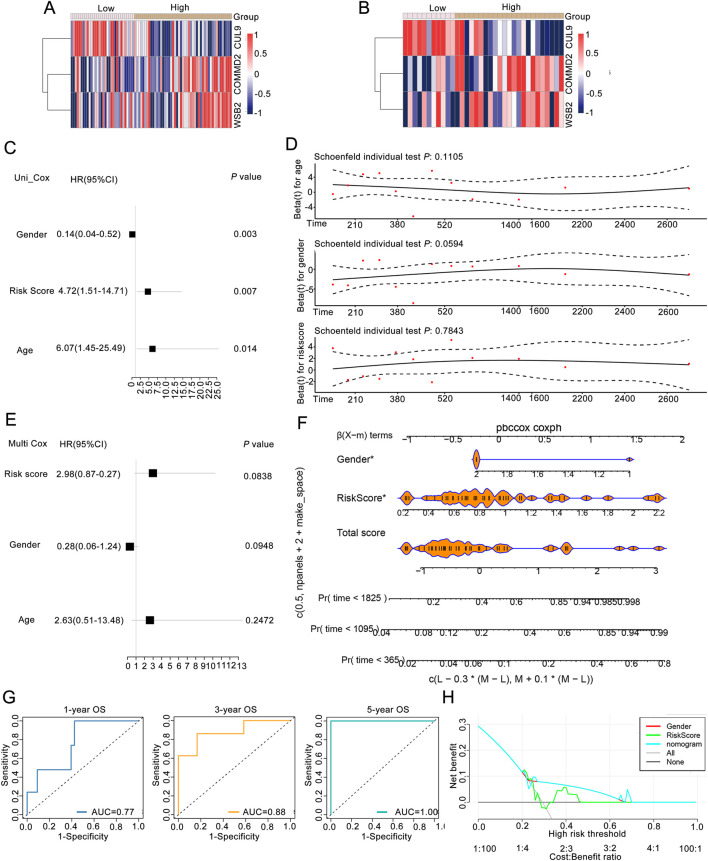
Association analysis between the risk score and clinical features of LSCC patients. **(A)** Expression of prognostic genes between groups of training set. **(B)** Expression levels of prognostic genes between groups in the validation set. **(C)** Univariate Cox regression analysis. **(D)** pH test analysis. **(E)** Prognostic factors screened by multivariate Cox analysis. **(F)** Nomogram of risk score in predicting OS. **(G)** ROC curves of risk score in predicting OS. **(H)** DCA curves of gender, riskscore, nomogram, and combined of them in predicting OS.

To explore risk score could be an independent prognostic factor, we integrated it with clinical characteristics (age, race, sex, PT, PM, PN, and tumor stage) in the training set. The expression heatmaps of *CUL9*, *COMMD2*, and *WSB2* in the training set and validation of the neddylation-associated prognostic signature are presented in Figures 3A,B. Univariate Cox regression and the PH assumption test were subsequently used to screen for independent prognostic factors. After excluding missing and uncertain data, we included 115 samples for analysis. The univariate Cox regression results demonstrated that age, sex, and the risk score were significant (*P* < 0.05; Figure 3C). The pH test confirmed that age, sex, and the risk score met the assumptions (*P* > 0.05; Figure 3D). Multivariate regression analysis further confirmed that the risk score was an independent prognostic factor for LSCC (*P* < 0.05; Figure 3E).

Moreover, to obtain reliable and convenient data for patient assessment, a nomogram model was constructed on the basis of the prognostic gene results to analyze the possible 1-, 3-, and 5-year survival rates of patients. The slopes of the calibration curves of the nomogram for 1-, 3-, and 5-year survival were close to 1, indicating that the constructed nomogram model has a certain degree of accuracy (Figure 3F). In addition, the prediction efficiency analyses suggested that all the AUC values were more than 0.6, indicating that the risk score of this signature performed well in predicting the 1-, 3-, and 5-year OS of LSCC patients (Figure 3G). Moreover, both the prognostic genes and the nomogram were above the All and None lines, indicating good clinical benefits (Figure 3H).

### GSEA of prognosis-associated gene signatures in LSCC

**FIGURE 4:**
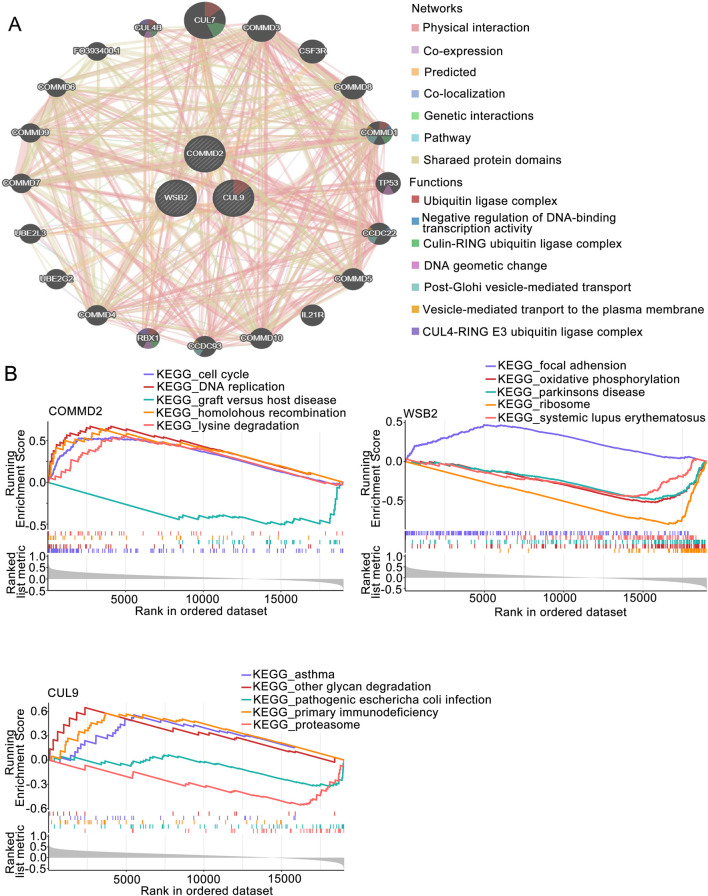
GSEA of the prognosis-associated gene signature. **(A)** GGI network of prognostic genes. **(B)** GSEA of key genes in the prognosis-associated gene signature.

The GGI of the prognostic genes was constructed on the GeneMANIA website. The top 20 correlations of these 3 prognostic genes and the top 7 significantly enriched pathways were subsequently selected for display (Figure 4A). As shown in the figure, genes such as *CUL9*, *COMMD2*, and *WSB2* were related to the prognostic genes. Pathways such as the ubiquitin ligase complex, negative regulation of DNA-binding transcription factor activity, and the cullin-RING ubiquitin ligase complex were related to the prognostic genes (Figure 4A). After performing GSEA on the prognostic genes, both the *CUL9* and *WSB2* genes were enriched in pathways such as the KEGG RIBOSOME (Figure 4B).

### Immune microenvironment analysis

**FIGURE 5:**
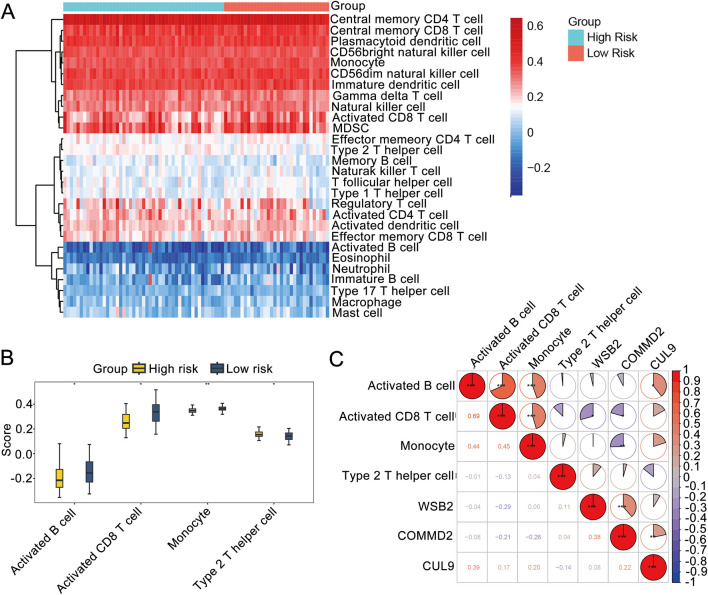
Immune microenvironment analysis. **(A)** Immune infiltration map. **(B)** Box plot of immune cell infiltration. **(C)** Correlations among prognostic genes, differential immune cells and differential immune cells.

To further understand the differences in immune cells infiltration in LSCC, ssGSEA was used to analyze the samples in the training set. The enrichment scores of 28 types of immune cells were obtained (Figure 5A). We also detected markedly differences in the immune infiltration percentages of 21 types of immune cells, including activated B cells, activated CD8 T cells, monocytes, and type 2 T helper cells, between the high- and low-risk groups (Figure 5B). Moreover, Spearman correlation analysis was carried out to further explore the correlations between the differential immune cells and the prognostic genes. As shown in Figure 5C, the differential immune cells with the highest correlations were activated B cells and activated CD8 T cells (R = 0.69, *P* < 0.05). WSB2 had the strongest negative correlation with activated CD8 T cells (R = −0.29, *P* < 0.05), and CUL9 had the strongest positive association with activated B cells (R = 0.39, *P* < 0.05).

### Drug sensitivity analysis and construction of a molecular regulatory network

**FIGURE 6:**
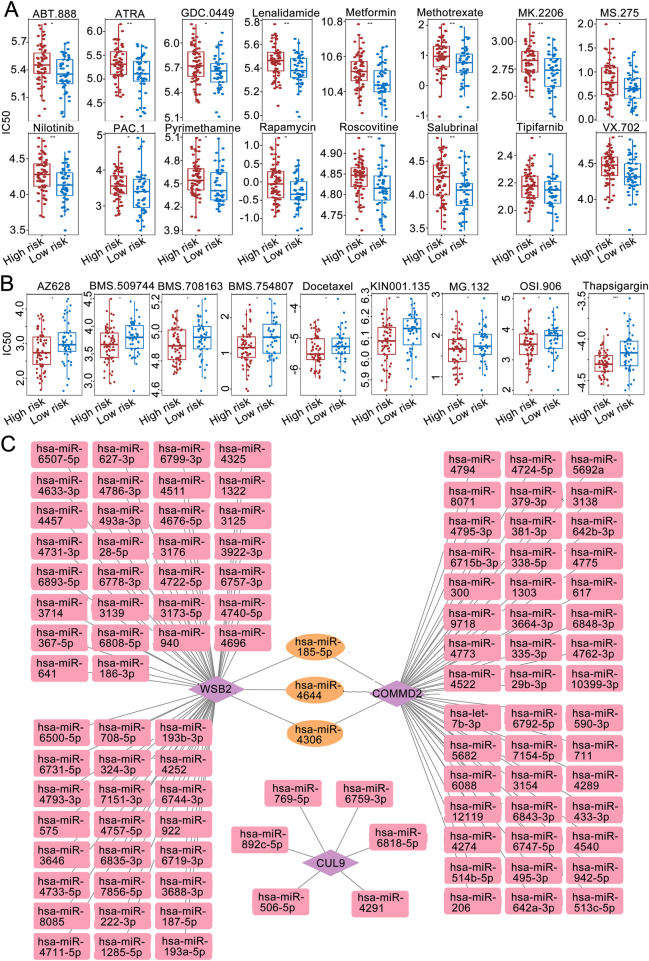
Immune microenvironment analysis. **(A)** IC50 sensitivity analysis of chemotherapy drugs among the high-risk groups. **(B)** IC50 sensitivity analysis of chemotherapy drugs among the low-risk groups. **(C)** miRNA‒mRNA regulatory network.

Drug sensitivity analysis revealed that among the high- and low-risk groups, the IC50 values of 16 drugs were significantly greater in the high-risk group than low-risk group (Figure 6A), and the IC50 values of 9 drugs were significantly lower in the high-risk group than low-risk group (*P* < 0.05; Figure 6B).

The miRWALK and miRDB databases jointly predicted the common miRNAs of the prognostic genes (Figure 6C). Among them, WSB2 was predicted to have 8 miRNAs in common in the databases. The targeted miRNA‒mRNA regulatory network diagram revealed a total of 9 nodes and 8 edges.

**FIGURE 7:**
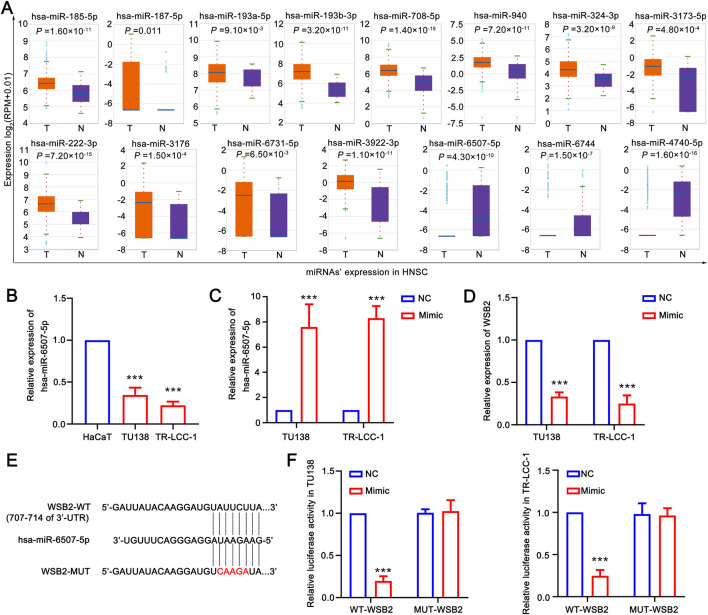
Hsa-miR-6507-5p might be upstream of WSB2. **(A)** Expression of miRNAs target WSB2 in HNSC in ENCORI database. **(B)** Expression of miR-6507-5p in LSCC cell lines. **(C)** Overexpression of has-miR-6507-5p in LSCC cell lines. **(D)** Expression of WSB2 in LSCC cell lines after overexpression of has-miR-6507-5p. **(E)** The binding sites between WT-WSB2/MUT-WSB2 and hsa-miR-6507-5p. **(F)** The regulatory relationship between hsa-miR-6507-5p and WSB2 confirmed using luciferase activity reporter assay. WT, wild type; MUT, mutant; ***P < 0.001.

To obtain LSCC associated DE-miRNAs, the expression of WSB2 specific associated miRNAs was further confirmed using the HNSC data in ENCORI database. A total of 15 differentially DE-miRNAs were screened out, and only 3 of them: hsa-miR-6507-5p, hsa-miR-6744-3p, and hsa-miR-4740-5p were significantly downregulated in HNSC, indicating 3 of them might be the upstream of WSB2 (Figure 7A). However, only qRT-PCR analysis showed that only hsa-miR-6507-5p was significantly downregulated in LSCC cell lines (Figure 7B), and the expression of has-miR-6744-3p and has-miR-4740-5p was hardly to detected due to their low expression. Hence, mimics of hsa-miR-6507-5p was transfected into TU138 and LR-TCC-1 and found that overexpression of hsa-miR-6507-5p mimics could significantly inhibit the expression of WSB2 (Figures 7C,D). Further luciferase activity reporter assay suggested that overexpression of hsa-miR-6507-5p mimics could markedly suppress the luciferase activity of WT-WSB2, but had no effect on the activity of MUT-WSB2 (Figures 7E,F). Taken together, these findings suggested that hsa-miR-6507-5p might be the upstream of WSB2 in LSCC.

### Silencing the key gene *WSB2* in the gene signature inhibited the malignant behaviors of LSCC

**FIGURE 8:**
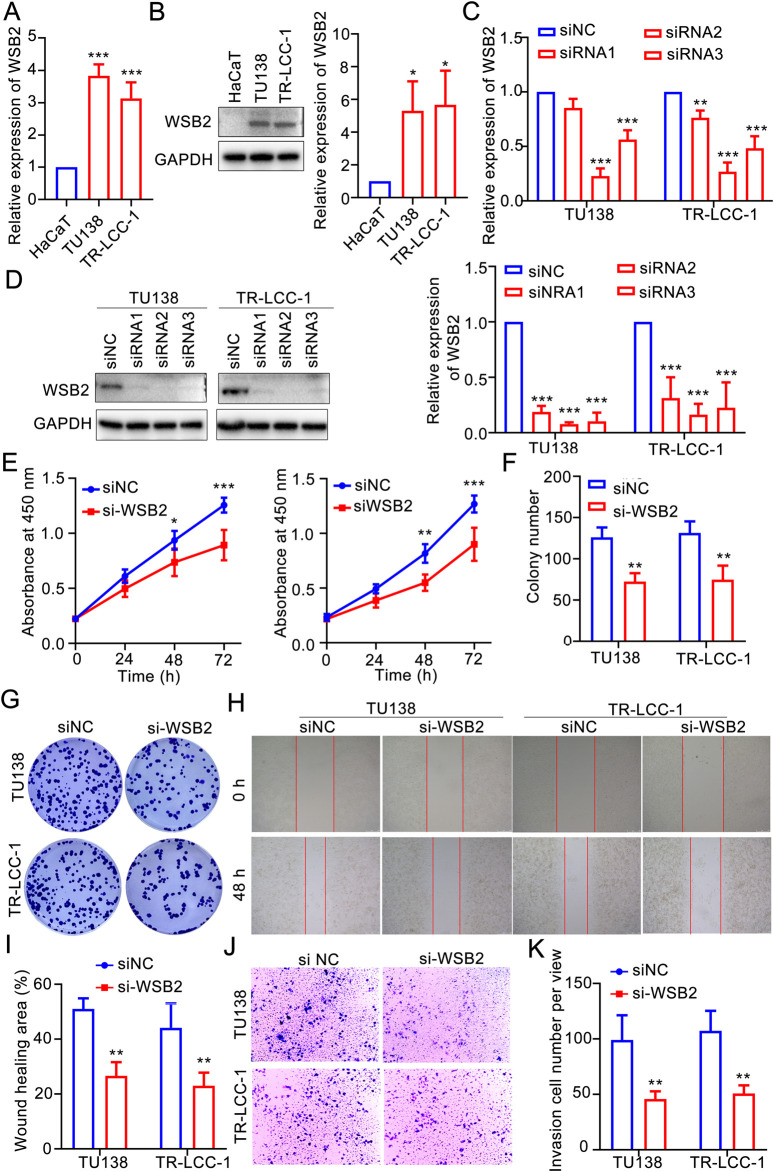
Silencing WSB2 inhibited the malignancy of LSCC. **(A)** Expression variation of WSB2 in LSCC. **(B)** Protein expression and quantification result of WSB2 in LSCC cell lines. **(C)** Confirmation of si-WSB2 via quantitative real-time PCR. **(D)** Confirmation of si-WSB2 via western blotting. **(E)** Proliferation of LSCC cells was detected via a CCK-8 assay. **(F)** Quantification of colony formation. **(G)** Colony formation ability of LSCC cells. **(H)** Migration of LSCC cells determined via wound healing. **(I)** Quantification of the wound healing assay results. **(J)** Invasion ability of LSCC cells determined via a transwell assay. **(K)** Quantification of the transwell assay results. *P < 0.05, **P < 0.01, and ***P < 0.001.

According to the aforementioned analyses, *COMMD2* and *WSB2* were two prognostic risk factors in the gene signature, and *COMMD2* had been previously reported in HNSCs (Tai et al., 2023). Thus, the role of WSB2 was explored in the present study. In comparison with HaCaT cells, expression of *WSB2* was markedly increased in the TU138 and LR-TCC-1 (Figures 8A,B). Accordingly, WSB2 was silenced in the TU138 and LR-TCC-1 cell lines via siRNA. Compared with siNC, siRNA2 and siRNA3 also significantly decreased the expression of WSB2 in both TU138 and LR-TCC-1 cells, and siRNA2 had the greatest effect on the inhibition of WSB2 (Figures 8B,C). Hence, siRNA2 was recorded as si-WSB2 and used for the following cell behavior experiments. The results of the CCK-8 assay suggested that, compared with siNC, si-WSB markedly decreased the proliferation ability of TU138 and LR-TCC-1 cells (Figure 8E). Like proliferation, si-WSB2 markedly inhibited the growth of TU138 and LR-TCC-1 cells compared with that in the siNC group (Figures 8F,G). Furthermore, wound healing and transwell analyses demonstrated that, compared with siNC, si-WSB2 obviously suppressed the migration and invasion capacity of TU138 and LR-TCC-1 cells (Figures 8H–K). These evidences suggested that silencing WSB2 expression markedly decreases the malignant behavior of LSCC cells, indicating that WSB2 could act as an underlying target in LSCC therapy.

## Discussion

In this study, 7,089 DEGs were identified through bioinformatics analysis, and 79 candidate genes were screened by taking the intersection with NRGs, which in turn led to the construction of a risk model identifying *COMMD2*, *WSB2* and *CUL9* as prognostic genes. The nomogram of this signature suggested greater accuracy in forecasting 1-, 3-, and 5-year OS. Hence, it is important to apply this gene signature in the clinical risk stratification of patients with LSCC, which might be beneficial for high-risk patients receiving neddylation-associated inhibitor treatment.

COMMD2, WSB2 and CUL9 contribute significantly to many cancers or tumors. Increasing evidence suggests that all COMMD family members play important roles in tumorigenesis and are expressed at higher levels in hepatocellular carcinoma (HCC) tissues than in normal tissues. The transcript level of COMMD2 was negatively correlated with OS. High COMMD2 expression is associated with tumor-induced activation of the immune response and immune infiltration in HCC (Wang et al., 2021). In addition, inhibition of COMMD2 suppressed the proliferation and migration of BLCA and uterine corpus endometrial cancer (UCEC) cells (Tai et al., 2023). WSB2 is an E3 ubiquitin ligase and also crucial in cellular cancers. A pan-cancer analysis has demonstrated that WSB2 is highly expressed in various cancers, and overexpression of WSB2 markedly promotes the proliferation and migration of breast cancer cells, which might depend on p53 signaling pathway (Deng et al., 2025). In HCC, elevated WSB2 expression degrades p53 and activates the IGFBP3-AKT-mTOR-dependent pathway, driving tumor development and metastasis. In addition, WSB2 also mediates the degradation of KLF15, result in a transcriptional repression of PDLIM2 and activation of NF-κB signaling pathway to promote development of HCC (Chen et al., 2025). In the present study, we also revealed that WSB2 was a prognostic risk factor in LSCC and that inhibiting WSB2 expression could significantly reduce the malignancy of LSCC cells. Moreover, WSB2 is a novel p53 destabilizer that promotes the polyubiquitination of K48-conjugated p53 at the Lys291 and Lys292 sites in HCC cells, leading to p53 proteasomal degradation (Li et al., 2024). In breast cancer, miR-28-5p inhibited breast cancer cell migration through the regulation of WSB2 (Ma et al., 2020), whereas in colorectal cancer (CRC), reduced expression of CUL9 has been reported to inhibit CRC cell growth (Zhang L. et al., 2024). CUL9, a potential p53-activated E3 ligase, promotes p53-dependent apoptosis (Pei et al., 2011) and is involved in immune response modulation in HNSCs (Xu et al., 2023). In addition, CUL9-mediated ubiquitination and degradation of Cytc constitute a strategy to alleviate apoptosis under mitochondrial stress in neurons and cancer cells (Gama et al., 2014). CUL9 also binds p53 to ubiquitinate heterogenous nuclear ribonucleoprotein C to inhibiting erastin-induced ferroptosis in CRC (Yang et al., 2022). Yes1 is considered as a key regulator of CUL9 phosphorylation at Y1505, while mutation of Yes1 or helicobacter-induced CUL9-Y1505 might switch CUL9 from a tumor-suppressor to an oncogene (Wu et al., 2023). While COMMD2, WSB2, and CUL9 have been implicated in various cancers, their roles in LSCC remain underexplored. Future research should focus on further verification and in-depth investigation of these genes in the context of LSCC.

GSEA revealed that the *CUL9* and *WSB2* genes were enriched in pathways such as KEGG RIBOSOME, highlighting their potential role in the regulation of protein synthesis. As a key site of protein synthesis, the proper function of ribosomes is crucial for cell growth, proliferation, differentiation and other processes, which are often abnormal during tumor development. The *CUL9* and *WSB2* genes may affect ribosome-related functions in various ways, which in turn are involved in the development of LSCC. Some genes can regulate ribosome assembly and function by affecting processes such as transcription and posttranslational modification of ribosomal proteins (Woolford and Baserga, 2013). Both CUL9 and WSB2 may regulate ribosomal activity, contributing to the development of LSCC through alterations in protein synthesis balance (Ruggero and Pandolfi, 2003).

Furthermore, we identified significant heterogeneity in the immune microenvironment of LSCC patients across different risk groups. Notably, significant differences were also identified in the immune cells’ infiltration, such as activated B cells and activated CD8^+^ T cells, were observed. These immune cells play important roles in tumor progression, with activated CD8^+^ T cells being the main effector cells of antitumor immunity. Greater infiltration of activated CD8 T cells is typically correlated with a better prognosis in various cancers, as they recognize and kill tumor cells (Rosenberg and Restifo, 2015). In contrast, dysfunctional or low-infiltrating CD8^+^ T cells may allow tumor cells to evade immune surveillance and promote metastasis. On the other hand, the role of activated B cells in tumor immunity is complex. While they can participate in humoral immunity by producing antibodies, they may also regulate immune responses by secreting cytokines or interacting with other immune cells (Shalapour and Karin, 2015). In this study, WSB2 was negatively associated with activated CD8 T cells, whereas CUL9 was positively associated with activated B cells, suggesting that these prognostic genes may influence LSCC prognosis by modulating immune cell function.

WSB2 was negatively associated with activated CD8 T cells, and CUL9 was positively associated with activated B cells. These correlations revealed that prognostic genes may affect the prognosis of LSCC patients via modulating immune cell function. Certain genes can influence CD8 T-cell function by regulating the expression of immune checkpoint molecules or cytokines (Pardoll, 2012). In melanoma, aberrant expression of a gene leads to the upregulation of immune checkpoint molecules, which inhibits the activation and killing function of CD8^+^ T cells and promotes the immune escape of tumor cells (Sade-Feldman et al., 2017). Owing to the positive interaction between CUL9 and activated B cells, CUL9 may enhance the humoral immune response by promoting processes such as activation, proliferation, or antibody secretion of activated B cells, which may affect tumor progression. However, relatively few studies have investigated the regulation of B-cell function by CUL9, and the specific molecular mechanisms involved need to be further explored.

Additionally, we identified specific miRNAs that may regulate WSB2 and COMMD2, including hsa-miR-185-5p, hsa-miR-4644, and hsa-miR-4306. These miRNAs are involved in the regulation of multiple cancers and may play crucial roles in the pathogenesis of LSCC by interacting with these genes. For example, hsa-miR-185-5p is a tumor suppressor in endometrial cancer (Oropeza-de Lara et al., 2024) and colon cancer, where it promotes migration and invasion by regulating IGF2 (Zhuang et al., 2020). In breast cancer, miR-185-5p inhibited cell proliferation by inducing apoptosis (Değerli et al., 2020). Similarly, miR-4644 has potential as a biomarker for pancreaticobiliary cancer (Machida et al., 2016) and bladder cancer treatment (Yan et al., 2020), whereas miR-4306 inhibits the proliferation of esophageal squamous cell carcinoma cells (Yang et al., 2021). These findings suggest that these miRNAs may influence LSCC progression by regulating tumor cell metabolism or immune microenvironment interactions. In this study, we had identified that hsa-miR-6705-5p might be served as a tumor suppressor in LSCC via downregulating WSB. A previous study suggested that lower expression of hsa-miR-6705-5p was markedly correlated with the poor prognosis of cervical cancer, which could inhibit the progression of cervical cancer via targeting CDK1 (Mei et al., 2020). Besides, hsa-miR-6507-5p was significantly downregulated in chronic sinusitis with nasal polyps, and might serve as a protective factor in the development nasal polyps via targeting NCAPG2 and PRC1 (Sun et al., 2022). These findings suggested that it is of significance to further clarify the role of hsa-miR-6507-5p/WSB2 in LSCC.

This study inevitably suffered some limitations. First, most of the results in this study were generated based on bioinformatic analysis but lack of validations due to different reasons, this might weak the reliability of conclusion. Second, although we had identified validate the role of WSB2 in LSCC, the role of WSB2 in neddylation of LSCC remains not further confirmed. Thus, a protein modification omics analysis will be performed to explore the downstream targets and underlying mechanisms of WSB2 in LSCC in our following investigation. Despite of these limitations, this study also provided us some direct evidences on the role of neddylation in LSCC.

## Conclusion

In this study, we successfully established an LSCC risk model based on COMMD2, WSB2, and CUL9. This model demonstrated good predictive efficacy in the training set, validation set and whole sample. Moreover, the risk score and sex were identified as independent prognostic factors, and the constructed nomogram had relatively high accuracy and favorable clinical benefits. Moreover, at the biological level, the enrichment characteristics of the DEGs were deeply dissected, the differences in immune cell infiltration between the high- and low-risk groups in the immune microenvironment and their correlations with prognostic genes were clarified, the differences in drug sensitivity were revealed, a molecular regulatory network was established, and diseases with relatively strong associations with key genes were identified. These findings may expand our understanding of the research and treatment of LSCC patients and provide new targets for LSCC.

## Data Availability

The datasets presented in this study can be found in online repositories. The names of the repository/repositories and accession number(s) can be found in the article/Supplementary Material.
