# Supplementary material for: Screening and regulatory mechanisms of biomarkers related to neddylation in laryngeal squamous cell carcinoma
Source: Front Mol Biosci. 2025 Oct 22;12:1654064. doi: 10.3389/fmolb.2025.1654064 (PMC12587159; doi:10.3389/fmolb.2025.1654064)
Supplement: Supplementary file 1 [file Supplementaryfile1.docx]

**Table S1 The sequences for siWSB2 and miR-6507-5p mimics**

| **Gene/miRNAs** | | **Sequences (5′→3′)** | |
| --- | --- | --- | --- |
| WSB2 | siRNA1 | Sense | GUCACGGCUUCUUACGAUA(dT)(dT) |
|  |  | Anti-sense | UAUCGUAAGAAGCCGUGAC(dT)(dT) |
|  | siRNA2 | Sense | GACAGUGACGUCCACAUUA(dT)(dT) |
|  |  | Anti-sense | UAAUGUGGACGUCACUGUC(dT)(dT) |
|  | siRNA3 | Sense | CCCUUCGAAGUUUCCUAACAA(dT)(dT) |
|  |  | Anti-sense | UUGUUAGGAAACUUCGAAGGG(dT)(dT) |
| hsa-miR-6507-5p | | Mimic | GAAGAAUAGGAGGGACUUUGU3 |
|  |  | NC | UUCUCCGAACGUGUCACGUTT |

**Table S2 The primers utilized in qRT-PCR**

| **Gene/miRNAs** | **Sequences (5′→3′)** | |
| --- | --- | --- |
| WSB2 | Forward | GTTAATTCGGAAGCTAGAGG |
|  | Reverse | CAAAGCCCATTGGTCATA |
| GAPDH | Forward | AGCCACATCGCTCAGACAC |
|  | Reverse | GCCCAATACGACCAAATCC |
| hsa-miR-6507-5p | Forward | AGGGAAGAATAGGAGGGACT |
|  | Reverse | CTTTGCATGGTACTGAACCA |
| U6 | Forward | CTCGCTTCGGCAGCACA |
|  | Reverse | AACGCTTCACGAATTTGCGT |
