# Supplementary figures and images for: Screening and regulatory mechanisms of biomarkers related to neddylation in laryngeal squamous cell carcinoma
Source: Front Mol Biosci. 2025 Oct 22;12:1654064. doi: 10.3389/fmolb.2025.1654064 (PMC12587159; doi:10.3389/fmolb.2025.1654064)

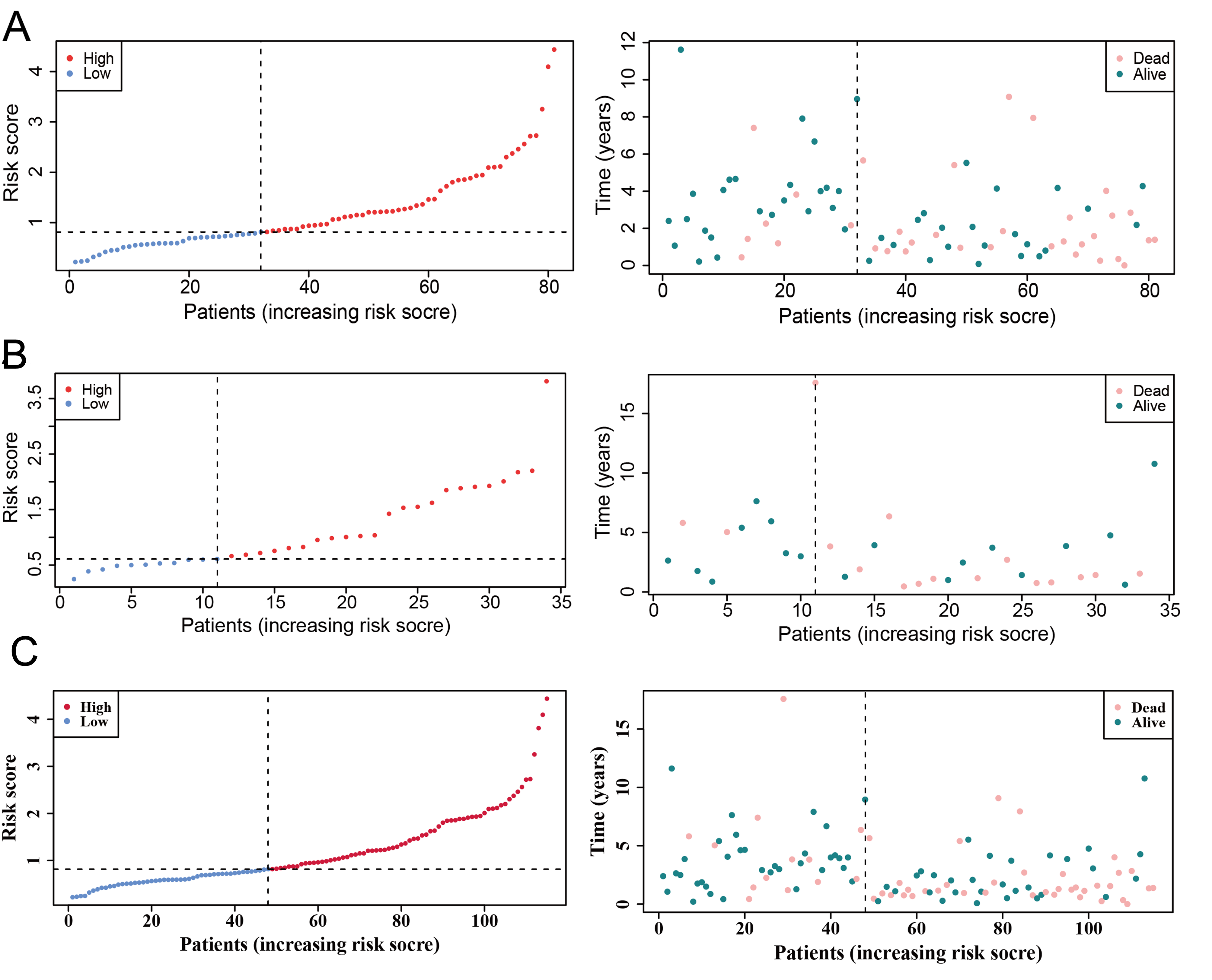

Supplement: Supplementary file 2 [file Image1.tif]
